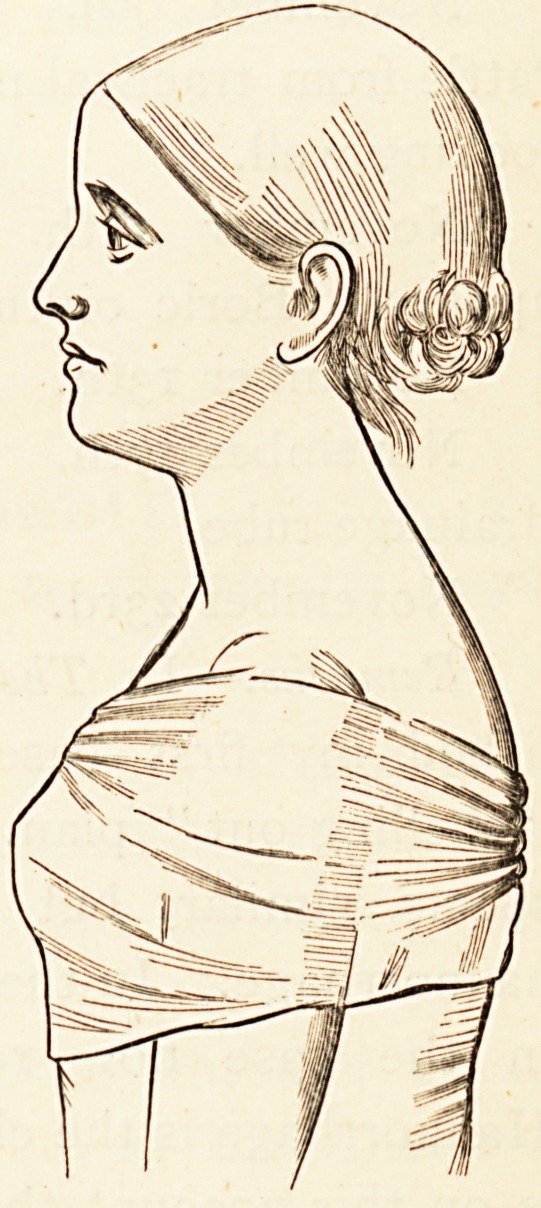# On a Case of Cystic Goitre Treated by "Shelling out"

**Published:** 1887-12

**Authors:** Charles Tanfield Vachell

**Affiliations:** Physician (formerly Surgeon) to the Cardiff Infirmary


### Clinical Records.

---

ON A CASE OF CYSTIC GOITRE TREATED BY "SHELLING OUT." By CHARLES TANFIELD VACHELL, M.D. Lond., Physician (formerly Surgeon) to the Cardiff Infirmary.

E. C., æt. 22, domestic servant, was admitted into the Cardiff Infirmary on October 19th, 1886, suffering from cystic tumour of the thyroid.

She first noticed the tumour about nine years ago, and since then it slowly and painlessly increased in size. Latterly it somewhat interfered with breathing, there being occasionally a choking feeling.

On admission the tumour was about as large as an orange, and occupied the front of the neck, inclining to the left side. It was painless, firm, and fairly movable, fluctuated distinctly, and rose and fell with deglutition. One rather large artery was felt entering at its right upper margin. An exploring needle was introduced, and it was found to be a single cyst and to contain fluid of a reddish colour.

It was decided to "shell out" the cyst, and on 27th October chloroform was administered, but the patient became so pallid that it was not considered advisable to proceed: four ounces of reddish fluid were then removed with the aspirator.

The tumour had quite filled again by 30th October; and on 3rd November, the patient having been etherised, an incision of about $2\frac{1}{2}$ inches in length was made through the skin, over the most prominent part, and the cyst was shelled out without difficulty.

There was free hæmorrhage from two vessels, which were readily secured, and this done, there was no further trouble in the operation. A drainage tube was inserted, and the wound dressed with carbolic gauze. The tumour weighed $11\frac{1}{2}$ oz., and contained $5\frac{1}{2}$ oz. fluid. It measured 9 inches in circumference, and was unilocular; the walls were from $\frac{1}{8}$ to $\frac{3}{16}$ of an inch thick.

November 6th. For some time after the operation the patient was much depressed; and during the night the wound was twice dressed, in consequence of oozing of bloody serum.

November 7th. Wound puffy, some swelling of surrounding tissues of neck, otherwise doing well.

November 8th. Wound dressed with carbolic oiled lint, and dressing frequently changed.

November 9th. Slight cough during night, slight rattle from tracheal mucus collecting in throat. Wound looking well.

November 11th. Sutures removed and strapping applied. Boric ointment dressing.

November 13th. To get up. Swelling subsided.

November 17th. Wound healed, except at position of drainage tube.

November 23rd. Discharged cured.

*Remarks.*—In *The Lancet* of 3rd July, 1886, is published my first case of cystic goitre treated by the "shelling out" plan. The two cases were in many respects similar, but differed chiefly in the amount of hæmorrhage. In the first case this was serious, whereas in the case now recorded it was easily controlled. Hæmorrhage is the chief danger of this operation, and it is on this account that Morell Mackenzie advocates the conversion of the cyst into a chronic abscess. In the *London Medical Record* for December 15th, 1884, appeared a note from an article by Dr. H. Burckhardt, of Stuttgart, in the *Centralblatt für Chirurgie*, No. 43, 1884, on "shelling out" of the tumour in cases of cystic goitre with a single cyst. The author concluded with a brief analysis of seventeen cases in which he had applied this operation, usually for dyspnœa. The treatment in each of these cases was attended with good results. A valuable paper by Dr. Morell Mackenzie was read before the Hunterian Society on 22nd January, 1872, in which the treatment by conversion into a chronic abscess was advocated in preference to extirpation; and recently (April 13th, 1887) Mr. T. Mark Hovell read a paper before the same Society supporting the same plan of treatment. Mr. Hovell is of opinion that "shelling out" exposes the patient to unnecessary risk, and mentions cases cured by him when the cysts contained 21 oz. and $10\frac{1}{2}$ oz. of fluid respectively.

## Figures and Tables

**Figure f1:**
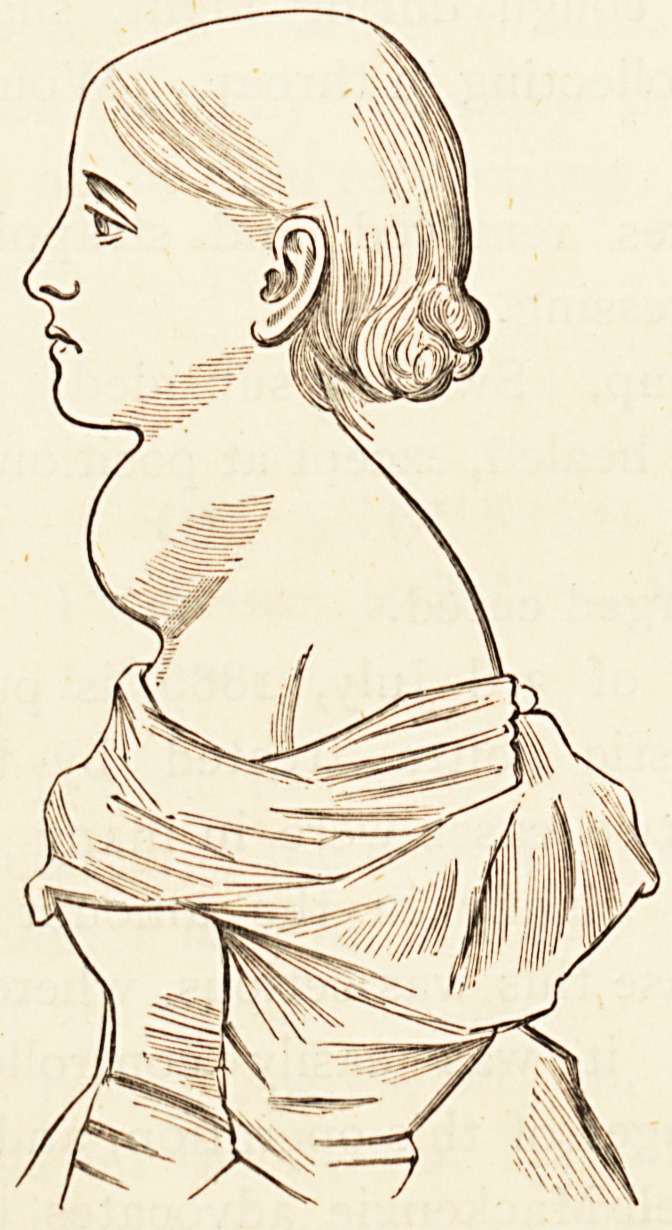


**Figure f2:**